# The intergenerational inequality of East Asian Economies under education premium: A comparative analysis based on ISSP2009

**DOI:** 10.1371/journal.pone.0337555

**Published:** 2026-01-28

**Authors:** Shouhao Li, Zhichen Lyu, Xinyi Gu

**Affiliations:** 1 School of Economics, University of Chinese Academy of Social Sciences, Beijing, China; 2 Center for Price Cost and Certification, National Development and Reform Commission, Beijing, China; 3 School of Education, Beijing Institute of Technology, Beijing, China; Visva-Bharati University: Visva-Bharati, INDIA

## Abstract

Recent studies have verified that the welfare regime is an important factor influencing intergenerational inequality, with the conservative regime being the most immobile one among the three welfare regimes adopted by western developed countries. Compared with the west, the productivist welfare regime is classified for East Asian economies due to their restrained welfare provision. This paper aims to explore the intergenerational inequality under productivist welfarism and finds that, under a productivist regime, individuals exhibit lower intergenerational inequality than those under a conservative regime, both in terms of father-to-child socioeconomic status and in the influence of fathers’ socioeconomic status on children’s income. Further analysis, grounded in human-capital theory, suggests that the education premium exists under a productivist regime is linked, albeit partially, to this finding.

## 1. Introduction

Intergenerational immobility has long been a central topic in the fields of economics, political science, and sociology. Over the past decades, scholars’ understanding has evolved from early concepts of market adjustment [1–2] to recognizing significant stratification patterns, as illustrated by the Great Gatsby Curve [3]. In *Capital in the Twenty-First Century*, Piketty [4] offers several highly pessimistic perspectives. First, he argues that intergenerational inequality is persistently expanding and that the post-World War II improvements were merely a temporary economic recovery following wartime destruction. Second, he identifies wealth inheritance as the dominant factor driving intergenerational inequality. Third, he posits that economic growth cannot outpace the return on capital, thereby rendering the redistributive power of income insufficient to counteract the solidifying effects of wealth inheritance on social stratification. Piketty’s assertions are not isolated and are supported by numerous scholars who provide additional empirical evidence and micro-level explanations. These studies largely attribute intergenerational immobility to structural factors, such as personal background. For instance, Chetty and Hendren [5–6] emphasize the role of community and occupational segregation, while Becker et al., Blanden and Macmillan, and Hai and Heckman [7–9] highlight education as a critical determinant in shaping human capital, which in turn determines social positioning. Similarly, research by Bianchi et al., Bjorklund and Jantti, Davis and Mazumder, and Pfeffer [ [10–13] underscores the influence of family background. Toft and Jarness, Jarness and Stromm, and Toft and Hansen [14–16] argue that upper-class parents utilize social networks to transfer advantages to their children. Meng and Li [17] reinforce these findings in their empirical work by comparing intergenerational inequality across different welfare regimes. But they only studied Western developed economies, where the welfare system was established on a relatively advanced stage of development, as these countries had mostly finished modernizing before WWII. As Piketty [4] points out, the post-war economic exuberance of Western economies was essentially a process of recovery. In this context, the experiences of Western economies may not be entirely applicable to the challenges faced by less developed nations today. It is therefore crucial to explore how welfare systems and intergenerational immobility evolve in other successful economies [18–19]. A natural focus would be East Asian economies, which are renowned for their “Asia’s Miracle” [20]. In the classification of welfare states, East Asia is often identified with the term “productivism” [21].

The productivist welfare regime, exemplified by East Asian economies such as Chinese Mainland, Japan, South Korea, Singapore, Hong Kong, and Taiwan district, emphasizes economic growth as its central objective. Holliday [21] argues that East Asian economies prioritize reinvestment over consumption to maximize productivity, a strategy shaped by their historical lag behind Western countries and their subsequent integration into globalization. In this global context, these economies have assumed the role of producing a wide array of goods primarily for Western developed markets. As a result, productivist welfare policies primarily aim to meet the basic needs of workers, ensuring their survival and sustained participation in the labor force. Midgley [22] characterizes this approach as “reluctant welfarism.” Several scholars have explored the distinctive features of East Asian welfare policies, including Goodman and Peng, and Ku and Jones Finer, among others [23–24]. There is broad consensus that the welfare policies of East Asian economies are designed to support economic growth while simultaneously solidifying social stratification [25–28]. In essence, access to welfare benefits is contingent upon active participation in the workforce, aligning individuals’ roles with the broader goals of economic growth. Consequently, welfare in productivist regimes is anchored to employment, echoing the conservative model’s insistence on tying benefits to work and social status. In short, productivist regimes make social spending conditional on boosting exports and jobs, so benefits stay lean and tied to work, whereas western models treat cash transfers, services and social insurance as citizenship rights that deliberately shield households from market shocks. That is to say, for redistribution, productivist is stingy while western Such unique features make productivist interesting for us to do a comparative study. Because productivist economies are on average economically less advanced, they might tend to generate sharper intergenerational inequality.

In this research, we include four East Asian economies—Chinese Mainland, South Korea, Japan, and Taiwan district—classified under the productivist regime, for a comparative analysis. As discussed earlier, the productivist regime represents a reluctant welfare model, where citizens do not have access to the extensive social protections available in Western welfare states. Similar to the conservative regime, identity plays a significant role in determining access to welfare under the productivist model. Based on this characteristic, we initially hypothesized that intergenerational inequality in East Asian economies would be more pronounced than in developed conservative economies. Surprisingly, the empirical evidence contradicts this expectation. A key factor to explain this outcome is education premium within fast growing economy.

The remainder of this article is organized as follows: Section 2 outlines the methods, hypotheses, and procedures. Section 3 details the data and variables used in the analysis. Section 4 presents the empirical findings, and Section 5 concludes with a discussion of the results.

## 2. Models and hypotheses

### 2.1. Models

This study utilizes data from the International Social Survey Programme – Social Inequality IV (ISSP2009). Since the ISSP2009 data is collected across various countries and regions, potential nonrandom sampling issues may arise. To address potential endogeneity caused by these sampling issues, we employ multilevel linear models to account for unobservable effects. The models comprise four equations. Equation (1) measures the impact of fathers’ International Socioeconomic Index of Occupational Status (ISEI) on children’ s ISEI, with a focus on how welfare regimes influence this relationship. Equation (2) examines the impact of fathers’ ISEI, welfare regime, and other relevant variables on the Income of children’s families. We emphasize income because it can largely characterize socioeconomic status and is a crucial outcome of human capital investment [29–30].

The equations are detailed as follows:


$$\begin{array}{cc} Respondent^{\prime }s\;{ISEI}_{ij}= & \;\beta_{\text{0}}+\beta_{\text{1}}*Father^{\prime }s\;{ISEI}_{ij}+\gamma_{\text{1}}*{Welfare\;Regime}_{j}+\eta_{\text{1}}*Father^{\prime }s\;{ISEI}_{ij}\\ & \times {Welfare\;Regime}_{j}+\beta *X_{ij}+\gamma *Z_{j}+U_{\text{0}j}+U_{\text{1}j}*Father^{\prime }s\;{ISEI}_{ij}+\varepsilon_{ij} \end{array}$$ (1)

where the dependent variable is the ISEI of respondent *i* from economy *j*, *Father’s ISEI*_*ij*_ corresponds to that respondent, and *Welfare Regime*_*j*_ is a series of dummy variables indicating to which welfare regime economy *j* belongs. The *η*_1_ measures the moderating effect from welfare regime to fathers’ impact on intergenerational mobility measured by *β*_1_. U_0*j*_ is the modifying components of hierarchical model to the intercept, and $U_{\text{1}j}$ is the random slope. *X*_*ij*_ represents individual-level controls and *Z*_*ij*_ represents economy-level controls, following the approaches outlined by existing studies [31–34].


$$\begin{array}{cc} Respondent^{\prime }s{\;Income}_{ij}= & \;\beta_{\text{0}}+\beta_{\text{1}}*{Father^{\prime }s\;ISEI}_{ij}+\gamma_{\text{1}}*{Welfare\;Regime}_{j}+\eta_{\text{1}}*Father^{\prime }s\;{ISEI}_{ij}\\ & \times {Welfare\;Regime}_{j}+\beta *X_{ij}+\gamma *Z_{j}+U_{\text{0}j}{+\;U}_{\text{1}j}*Father^{\prime }s\;{ISEI}_{ij}+\varepsilon_{ij}\;\;\;\;\;\;\;\;\;\;\;\;\;\;\;\;\;\;\;\;\;\; \end{array}$$ (2)

where the dependent variable is the personal income or family income of respondent *i* from economy *j*, and the main independent variables are *Fathers’ ISEI,* and *Welfare Regime*. Still *η*_1_ tells us how welfare regimes moderate fathers’ ISEI. Other variables are the same as (1). As ISEI, income is meanwhile a heavily researched indicator when it comes to intergenerational inequality. The logic is that the long-established welfare provision eases household budgets, allowing slightly more spending on schooling, and then schooling turns into human capital and finally income [7,8,35].

Finally, in order to compare the coefficients of independent variables as well as different models, we run standardized regression in the empirical section, and we employ the sampling weight of ISSP in most models.

### 2.2. Hypotheses

Based on the considerations presented in section 2.1, we propose the following null hypotheses. First, the socioeconomic status of individuals in productivist economies is more strongly influenced by their fathers’ socioeconomic status. This hypothesis is based on the premise that productivism, as a reluctant welfare regime, provides minimal welfare benefits to its citizens. The variable of interest for testing this hypothesis is to $\eta_{\text{1}}$ in equation (1). Using conservative regime as reference group, we set H1 as $\eta_{\text{1}}$ = 0 and we expect to observe a positive significant result for productivist.

Second, the income of individuals in productivist economies is more significantly affected by their fathers’ socioeconomic status. As in H1, we set H2 as $\eta_{\text{1}}$ =0 in equation (2) and we expect to observe a positive significant result for productivist.

## 3. The details of data and variables

### 3.1. The details of data

The primary dataset used in this study is retrieved from the International Social Survey Programme – Social Inequality IV (ISSP2009), which provides cross-sectional, individual-level data. Due to the hierarchical structure of the data, ignoring correlated errors could result in biased estimates. To address this, we employ a hierarchical linear model. And to compare the magnitudes of regression coefficients for key variables, we standardize non-dummy variables using z-scores, following the recommendations of Gelman and Hill [36].

This study examines 20 economies categorized into four welfare regime types. The liberal group includes Australia, New Zealand, the U.K., and the U.S.; the conservative group comprises Austria, Belgium, France, Germany, Italy, Portugal, Spain, and Switzerland; the Nordic group contains Denmark, Finland, Norway, and Sweden; and the productivist group includes Chinese Mainland, Japan, South Korea, and Taiwan district. Since the ISSP2009 dataset does not include data for Hong Kong and Singapore, the analysis focuses on four economies within the productivist welfare regime: Chinese Mainland, Japan, South Korea, and Taiwan district. Although the ISSP survey covers additional economies, they are excluded either due to missing data for key variables, or lack of alignment with the four welfare regimes of interest.

### 3.2. The construction of variables

The two primary dependent variables in our model are occupation status and income. Occupation status, which serves as a proxy for socioeconomic status, is measured using the International Socioeconomic Index of Occupational Status (ISEI). Following the methodology of Breen et al. [37], we employ the algorithm developed by Ganzeboom et al., and Ganzeboom and Treiman [38–39] to calculate the ISEI rankings for respondents. Although the ISEI measure may face challenges related to contextualization across different economies, it has been demonstrated to be robust in international comparative research [38–39]. By using ISEI, we address the limitation of relying solely on pure economic indicators such as income, which do not account for the endowments of children [40].

To calculate the paternal ISEI (father’s ISEI), we use the response to question V57 - Q15b of the ISSP 2009 questionnaire: “*When you were <14/15/16> years old, what kind of work did your father do?*” Similarly, the respondent’s ISEI is calculated using their answer to question Q19b of the ISSP: “*In your current job, what is your main occupation?*” By employing this approach, we convert occupational data from discrete categories into a continuous variable.

To measure income, we employ four proxies. The first is respondents’ own individual income ranks and the Second is respondent’s family income ranks. Both of them are from ISSP 2009 questionnaire. Family income could not only play as a role of robust analysis but also is a newly researched indicator for intergenerational inequality [41]. Finally, two composite income factors are calculated by SEM model with individual income, family income, education years, and education degree as inputs, providing a latent measure of income.

The core explanatory variable in this study is the welfare regime type, categorized into four types: liberal, conservative, Nordic, and productivist. This classification aligns our research sample with previous studies, such as Hertel and Groh-Samberg [42], and Meng and Li [17], which focus on Western countries and adopt Esping-Andersen’s three-regime framework [43].

For controls, at the individual level, they include gender, age, and education information. At the economy level, we match each respondent with GDP per capita (PPP) when he or she was 15 year-old. Because respondents span many birth years, early macro controls are often missing, so to keep sample attrition low we retain only 5-year average GDP per capital centered at age 15. Detailed descriptions of all variables used in the analysis are presented in Table 1.

**Table 1:** The vocabulary of variables.

Name	Meaning	Attribute	Database
Age	respondent’s age	Individual	ISSP2009
Gender	respondent’s Gender	Individual	ISSP2009
Income	Individual income	Individual	ISSP2009
Family income	Family income	Individual	ISSP2009
Education	respondent’s education years	Individual	ISSP2009
Degree	respondent’s academic diploma rank	Individual	ISSP2009
Fathers’ ISEI	the socio-economic status of respondent’s father, coded according to Ganzeboom et al. (1992)	Individual	ISSP2009
R-ISEI	the socio-economic status of respondents, coded according to Ganzeboom et al. (1992)	Individual	ISSP2009
GDP per capita	5-year average GDP per capita (PPP) centered at 15-year-old of respondents	Economy	Penn World Table.
Liberal, Conservative, Nordic, Productivist.	Regime type dummy variables	Economy	Esping-Andersn (1990), and Holiday (2001).

Note: We use standardized variables except dummy variables. Income variables are firstly ranked in each economy to avoid improper comparison.

## 4. Empirical results

### 4.1. Baseline empirical results and the contradiction of productivism

To analyze empirical relationships, we first examine how welfare regime types influence intergenerational inequality corresponding to equation (1) in section 2.1. Table 2 presents the baseline empirical results, showing how fathers’ ISEI impacts their children’s ISEI across the four regime types, with conservatism serving as the reference group. The dummy variables “Liberal”, “Nordic”, and “Productivist” indicate whether respondents originate from economies belonging to these respective regimes.

**Table 2:** The empirical results of four regime types.

	(1)	(2)	(3)	(4)
VARIABLES	R-ISEI	R-ISEI	R-ISEI	R-ISEI
Father’s ISEI	0.405***	0.231***	0.228***	0.234***
	(0.016)	(0.011)	(0.011)	(0.019)
Age		0.094***	0.109***	0.134***
		(0.007)	(0.016)	(0.034)
Gender		−0.102***	−0.109***	−0.124***
		(0.012)	(0.013)	(0.017)
Education		0.332***	0.334***	0.334***
		(0.007)	(0.008)	(0.045)
Income		0.315***	0.320***	0.321***
		(0.007)	(0.007)	(0.024)
GDP per capita			0.049*	0.085
			(0.025)	(0.061)
Liberal	0.103	0.032	0.012	0.010
	(0.080)	(0.056)	(0.058)	(0.060)
Nordic	0.126	0.081	0.066	0.082
	(0.080)	(0.056)	(0.058)	(0.050)
Productivist	−0.206***	−0.179***	−0.119*	−0.094
	(0.079)	(0.056)	(0.067)	(0.121)
Liberal*Father’s ISEI	−0.181***	−0.102***	−0.098***	−0.112***
	(0.028)	(0.017)	(0.018)	(0.021)
q value				0.000
Nordic*Father’s ISEI	−0.160***	−0.085***	−0.082***	−0.087***
	(0.028)	(0.017)	(0.018)	(0.018)
q value				0.000
Productivist*Father’s ISEI	−0.105***	−0.060***	−0.061***	−0.064***
	(0.027)	(0.016)	(0.016)	(0.023)
q value				0.016
Constant	0.034	0.039	0.026	0.001
	(0.046)	(0.033)	(0.035)	(0.045)
ISSP WEIGHT	NO	NO	NO	YES
Robust standard error	NO	NO	NO	YES
Inter-class correlation (ICC)	1.88%	1.17%	1.23%	1.43%
Correlation random beta & slope	negative	negative	negative	negative
BIC	56600.75	44788.72	42463.8	42014.44
Observations	21,257	18,641	17,675	17,675
Number of groups	20	20	20	20

Standard errors in parentheses, *** p < 0.01, ** p < 0.05, * p < 0.1

Across the four models, with more controlling variables gradually added into, the interaction terms between the three regime dummies and fathers’ ISEI are consistently significant and negative, and the q value [44] keeps below 0.05, not supporting the possibility of false discovery. This finding indicates that, even in comparison with the productivist regime, the conservative regime exhibits the highest degree of intergenerational inequality. However, among the three cross product terms, productivism demonstrates the weakest attenuation of fathers’ ISEI influence on children’s ISEI. Nevertheless, compared to conservatism, the productivist regime does reduce the transmission of intergenerational inequality. So the findings of productivism is mixed.

Therefore, Hypothesis 1 is partially supported: while productivism mitigates the influence of fathers’ ISEI on children’s ISEI relative to conservatism, it is less effective in doing so compared to the liberal and Nordic regimes. Among other independent variables, education and income are the most important ones to determine respondents’ ISEI. Although Models (1) and (2) cannot be compared via BIC because their samples differ, comparing BIC between Models (3) and (4) is unproblematic. Then we see a smaller BIC value for model (4) which relies on ISSP sampling weights and robust standard error. Thus we continue with ISSP weights and robust standard errors for following empirical analysis.

**Fig 1:**
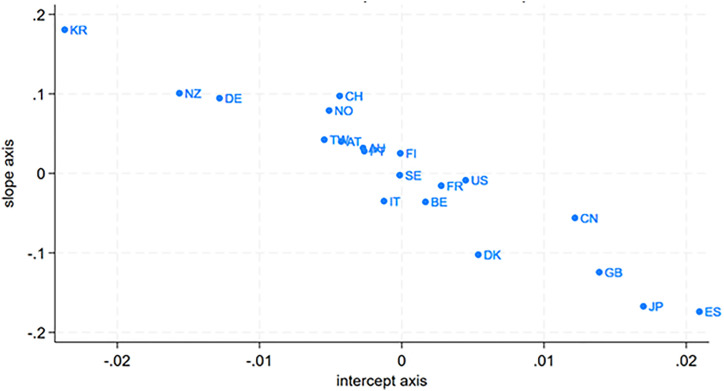
The Scatter Plot of Random Intercept with Random Slope.

**Fig 2:**
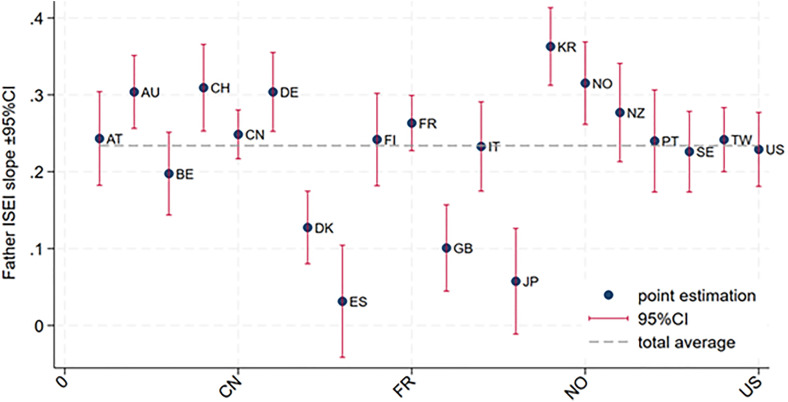
The 95% Interval of Intergenerational ISEI Link across Economies.

Meanwhile, we observe a negative correlation between the random slope and intercept in Table 1, which means economies with higher children’s ISEI than global average will have less degree of intergeneration immobility. The inter-class correlation stays below 2%, not that large but worth discussing. So next, Fig 1 shows the scatter plot of the random slope and random intercept of equation (1) according to the calculation of model (4) of Table 2. Mainly the Nordic group and conservative group gather in the middle, while members of liberal or productivist group spread with dispersion. Fig 2 plots the overall coefficient of intergenerational ISEI—that is, the sum of the estimate for fathers’ ISEI and the mean random slope from equation (1)—together with its confidence interval. It is evident that the Nordic group mostly lies below the overall average, indicating the mildest intergenerational inequality, while members of the other three regimes exhibit considerable heterogeneity. This finding indicates that, after partialing out welfare regime, there remain substantial differences across regions. And of course, the negative cross product in Table 2 will drag down the members except of conservative group to a considerable extent.

Table 3 presents the robust regressions supplementary for Table 2. When collecting information on fathers’ occupations, ISSP 2009 did not use ISCO-88 codes for the United Kingdom and New Zealand; instead, it employed a simplified one-digit occupational classification. We mapped these one-digit codes to the corresponding ISCO-88 major groups and therefore assigned all fathers from these two countries the mean ISEI score of their respective ISCO-88 major group. This approximation may bias the results, so Model (1) in Table 3 excludes UK and New Zealand. The findings are virtually unchanged to those reported in Table 2. Model (2) adopts a spline method [45] and model (3) uses the ISEI ranks of respondents as well as their fathers as the inputs for spline model with three piecewise linear parts. The p-value of 0.02 of LR test confirms the existence of nonlinearity. As we can see, for the productivist group, there is a stratified intergenerational ISEI relation. For the lower and middle classes, there are suppressive and amplifying effects for this ISEI relation. respectively when using the rank model. For both models, the upper class shows a suppressive effect.

**Table 3:** Robust analysis of the four regime types.

	(1)	(2)	(3)
	UK/NZ Deleted	Spline Model	Rank Spline Model
VARIABLES	R-ISEI	R-ISEI	R-ISEI
Father’s ISEI	0.236***		
	(0.018)		
Father’s ISEI _sp1		0.351***	0.260***
		(0.065)	(0.046)
Father’s ISEI _sp2		0.218***	0.113***
		(0.043)	(0.040)
Father’s ISEI _sp3		0.212***	0.364***
		(0.024)	(0.046)
Liberal	0.023	−0.088	0.030
	(0.038)	(0.107)	(0.039)
Nordic	0.085*	−0.037	0.053
	(0.050)	(0.113)	(0.036)
Productivist	−0.085	−0.087	0.123
	(0.124)	(0.096)	(0.108)
Liberal*Father’s ISEI	−0.101***		
	(0.016)		
Nordic*Father’s ISEI	−0.088***		
	(0.017)		
Productivist*Father’s ISEI	−0.064***		
	(0.023)		
Liberal*Father’s ISEI_sp1		−0.227*	−0.219*
		(0.122)	(0.118)
Nordic*Father’s ISEI_sp1		−0.231*	−0.183
		(0.131)	(0.113)
Productivist*Father’s ISEI_sp1		−0.026	−0.466***
		(0.084)	(0.119)
Liberal*Father’s ISEI_sp2		−0.031	0.080
		(0.081)	(0.073)
Nordic*Father’s ISEI_sp2		−0.070	−0.006
		(0.075)	(0.058)
Productivist*Father’s ISEI_sp2		−0.061	0.202***
		(0.069)	(0.078)
Liberal*Father’s ISEI_sp3		−0.111***	−0.287***
		(0.040)	(0.056)
Nordic*Father’s ISEI_sp3		−0.062	−0.136
		(0.038)	(0.092)
Productivist*Father’s ISEI_sp3		−0.113***	−0.278***
		(0.042)	(0.077)
Constant	−0.005	0.117*	0.308***
	(0.045)	(0.060)	(0.026)
Controls	YES	YES	YES
ISSP WEIGHT	YES	YES	YES
LR test p value	NA	0.02	NA
Observations	16,417	17,675	17,675
Number of groups	18	20	20

Robust standard errors in parentheses, *** p < 0.01, ** p < 0.05, * p < 0.1

Table 4 presents the estimates for four alternative measures of respondent’s income: personal income, family income, and two latent income factors extracted by SEM. The first latent factor is based only on personal and family income (α = .77), whereas the second additionally incorporates years of schooling and diploma attainment (α = .76). Both latent factors achieve the hurdle of Cronbach α. All four specifications correspond to Equation (2) shown in section 2.1. For the results, father’s ISEI exerts a strong and highly significant positive effect on every income measure, underscoring the persistent importance of parental socioeconomic status. As for our main concern, seldom do the interaction terms achieve consistent significance across the four models; thus the hypothesis 2 seems not to be rejected. Nevertheless, the productivist regime dummy is positively signed and statistically significant in every equation, indicating a systematic income premium within productivist economies that merits more investigation.

**Table 4:** The impact of fathers’ ISEI to respondents’ income.

	(1)	(2)	(3)	(4)
VARIABLES	Income	Family income	Income factor1	Income factor2
Father’s ISEI	0.053***	0.083***	0.074***	0.044***
	(0.020)	(0.022)	(0.023)	(0.010)
Age	0.262***	−0.065	−0.052	−0.002
	(0.084)	(0.042)	(0.039)	(0.018)
Gender	0.524***	−0.188***	−0.182***	−0.028***
	(0.037)	(0.025)	(0.024)	(0.008)
Education	0.290***	0.099***	0.095***	0.310***
	(0.030)	(0.027)	(0.026)	(0.029)
Income		0.571***	0.577***	0.110***
		(0.023)	(0.021)	(0.008)
GDP per capita	0.239**	0.102***	0.106***	0.060**
	(0.116)	(0.038)	(0.037)	(0.025)
Liberal	−0.016	−0.039	−0.031	0.057
	(0.082)	(0.031)	(0.030)	(0.035)
Nordic	−0.070	−0.085**	−0.076**	0.071***
	(0.074)	(0.040)	(0.039)	(0.018)
Productivist	0.364*	0.147**	0.162**	0.177***
	(0.216)	(0.068)	(0.069)	(0.041)
Liberal*Father’s ISEI	0.029	0.018	0.021	0.014
	(0.030)	(0.023)	(0.024)	(0.013)
Nordic*Father’s ISEI	0.018	−0.055**	−0.046*	−0.017
	(0.027)	(0.023)	(0.024)	(0.013)
Productivist*Father’s ISEI	0.014	0.029	0.033	−0.005
	(0.033)	(0.025)	(0.025)	(0.007)
Constant	−0.310***	0.093***	0.097***	−0.058***
	(0.089)	(0.033)	(0.031)	(0.015)
ISSP WEIGHT	YES	YES	YES	YES
Average inter-item correlation	NA	NA	0.60	0.44
Cronbach α	NA	NA	0.75	0.76
Observations	20056	19,228	20,056	20,056
Number of groups	20	20	20	20

Robust standard errors in parentheses, *** p < 0.01, ** p < 0.05, * p < 0.1

## 5. Discussion of the empirical findings

### 5.1. Facts about East Asian economies

In the previous section, we observe a strong intergenerational relation between fathers’ socioeconomic status and children’s. Although the productivist regime visibly weakens the direct transmission of occupational status (ISEI) across generations, it seems not to dilute the payoff of fathers’ ISEI on children’s income. We think it is necessary to explore additional details related to the nature of welfare regimes. The regime of primary interest in this study is productivism, which is fundamentally centered on promoting economic growth under the background of globalization that transfer industry to East Asian areas. A natural outcome of such trend is industry upgradation which needs labor with more human capitals [46–47]. For instance, from 1998 to 2009, the college admission of China enlarged 5 times [48], and private investments to education keeps at high level [49]. The fact is that the reward to education is in premium [48,50–52]. Thus, we propose that to understand the story behind income lies in incorporating education into the models. Within this framing, the existing human-capital theory of education [8,9,29,30] fits naturally. Human capital theory posits that education enhances individual productivity by equipping learners with skills and knowledge, thereby increasing their marginal revenue product and wages. This aligns with East Asia’s emphasis on mass education as a driver of rapid industrialization. For instance, state-led investments in tertiary education amplified the supply of high-skilled labor, meeting the demands of technology-intensive industries during the region’s industrial upgrading phase.

### 5.2. Education premium with shift of industry

Table 5 presents the results when education and income are jointly analyzed. In Model (1), we observe a significant positive link between respondents’ education and productivist regime, as well as Nordic. Moving to model (2), it is evident that educational level has a substantial and statistically significant positive effect on an individual income. Concurrently, the interaction term “Father’s ISEI*Education” shows a negative and significant coefficient. This indicates that longer duration of education serves to weaken the influence of the father’s ISEI on respondents’ income. When family income is substituted for personal income in Model (3), the pattern of interaction term replicates that of model (2). Taken together, Table 5 shows that productivism correlates positively to education which raises respondents’ income. Thus by the logic shown in section 5.1, it is the education premium that attenuates the effect of fathers’ ISEI to children’s income.

**Table 5:** The discussion about education I.

	(1)	(2)	(3)
VARIABLES	Education	Income	Family income
Father’s ISEI	0.212***	0.070***	0.087***
	(0.024)	(0.012)	(0.010)
Age	−0.042	0.252***	−0.074*
	(0.038)	(0.082)	(0.039)
Gender	−0.070*	0.523***	−0.189***
	(0.037)	(0.038)	(0.026)
Education		0.290***	0.098***
		(0.029)	(0.026)
Income	0.212***		0.571***
	(0.016)		(0.023)
Family income			
GDP per capita	0.319***	0.218**	0.082*
	(0.060)	(0.110)	(0.042)
Liberal	0.163		
	(0.103)		
Nordic	0.275**		
	(0.117)		
Productivist	0.309*		
	(0.178)		
Father’ ISEI*Education		−0.017*	−0.017*
		(0.010)	(0.010)
Constant	−0.117	−0.246***	0.106***
	(0.087)	(0.066)	(0.033)
ISSP WEIGHT	YES	YES	YES
Observations	20,056	20,056	19,228
Number of groups	20	20	20

Robust standard errors in parentheses, *** p < 0.01, ** p < 0.05, * p < 0.1

Table 6 presents regression results by replacing education with respondents’ academic degree as dependent variable. In Model (1), the productivist regime shows its benign relation with respondents’ academic degrees, as evidenced by its significant coefficient. Models (2) and (3) examine how respondents’ academic degrees affect their income, using individual earnings in (2) and family income in (3). Surprisingly, the degree dummy is small and insignificant in (2), whereas it attracts a large, highly significant coefficient in (3).

**Table 6:** The discussion about education II.

	(1)	(2)	(3)
VARIABLES	Degree	Income	Family income
Father’s ISEI	0.261***	0.019*	0.071***
	(0.018)	(0.010)	(0.010)
Age	−0.025	0.239***	−0.069*
	(0.072)	(0.075)	(0.038)
Gender	−0.112***	0.519***	−0.183***
	(0.043)	(0.036)	(0.028)
Degree		0.386***	0.146***
		(0.014)	(0.021)
Income	0.304***		0.549***
	(0.017)		(0.022)
Family income			
GDP per capita	0.375***	0.163*	0.067*
	(0.095)	(0.098)	(0.037)
Liberal	0.261**		
	(0.132)		
Nordic	0.291***		
	(0.081)		
Productivist	0.696***		
	(0.227)		
Father’ ISEI*Degree		0.010	−0.023***
		(0.011)	(0.009)
Constant	−0.244**	−0.237***	0.110***
	(0.099)	(0.058)	(0.032)
ISSP WEIGHT	YES	YES	YES
Observations	20,315	20,315	19,475
Number of groups	20	20	20

Robust standard errors in parentheses, *** p < 0.01, ** p < 0.05, * p < 0.1

Overall, the results in Tables 5 and 6 lend some support to the conjecture that, under globalization, the eastward shift of industry to East Asia has generated an education premium which allows productivist respondents to translate education into higher income. These findings are in line with the work of other scholars [52–54]. Yet, in the sense of significance, the evidence remains partial.

## 6. Conclusion

This study investigates intergenerational inequality in East Asian economies under the productivist welfare regime [21], an addition to Esping-Andersen’s [43] three Western welfare state models. The defining feature of productivism is its provision of less comprehensive welfare benefits compared to the Western countries analyzed by Esping-Andersen. Based on this, we hypothesized that productivist economies would exhibit greater intergenerational immobility than their Western counterparts. However, our empirical evidence reveals a contrasting narrative. Productivist economies demonstrate lower levels of intergenerational inequality compared to conservative welfare regime whose members are developed economies primarily located in Western Europe. This study makes several contributions to academic literature:

First, international comparisons of intergenerational inequality. This paper answers the calls of Fox et al. and Bratberg et al. [55–56] for more comparative studies on intergenerational inequality. By incorporating East Asian economies, it broadens the scope of analysis beyond Esping-Andersen’s original framework and highlights the unique role of productivism in mitigating inequality.

Second, insights into productivist economies. Our findings complement the research of Meng and Li [17] on intergenerational inequality within Esping-Andersen’s three classical welfare regimes. By comparing productivist economies with Nordic, liberal, and conservative regimes, we show that productivism ranks below Nordic and liberal regimes but achieves lower intergenerational inequality than conservatism, despite its reluctance to provide extensive welfare benefits.

Third, education premium under the shift of industry. This study underscores the centrality of education premium, the elevated economic returns to higher educational attainment, in mitigating intergenerational inequality within East Asian productivist regimes. These findings are actually rooted in human capital theory [29–30]and signaling mechanisms [57]. East Asian economies limit welfare provision and chase economic growth, and thus their people keep high saving rate which in turn promotes growth again [58–59]. With the pace of industry shift to east, these economies have historically experienced higher levels of economic growth and hence require more human capital [48,54]. Meanwhile they invest large amount of resources in education, so do their households [60], then the younger people benefit from education premium.

If welfare provision is essential for ensuring societal stability and human rights, then the productivist model presents a reference for developing economies aiming to maintain economic growth while avoiding the trap of entrenched intergenerational inequality. By prioritizing skill formation over redistribution, productivist regimes demonstrate that targeted educational expansion aligned with industrial needs can offset inequality without sacrificing growth. For emerging economies currently with high growth such as Vietnam, Rwanda, and Ethiopia, this offers a viable path to balance equity and development.

We frankly admit the limitations of our study. This research takes a broad, cross-economy view, so it cannot unpack the fine-grained differences that separate welfare institutions inside each economy. Because most variables are built from survey answers, measurement error is unavoidable, and the macro level controlling variables are not various. And the empirical results about income are especially fragile. Future work should pick a few typical economies and compare their micro-level settings in greater detail. Such work can use panel social-survey data from interested economy if available to capture change over time.

## Supporting information

S1 Table Descriptive Statistics. The table downward shows the descriptive statistics (Mean and N) of the variables used.
(DOCX)

S1 File Stata codes and files. (ZIP)
