# Supplementary material for: The intergenerational inequality of East Asian Economies under education premium: A comparative analysis based on ISSP2009
Source: PLoS One. 2026 Jan 28;21(1):e0337555. doi: 10.1371/journal.pone.0337555 (PMC12851476; doi:10.1371/journal.pone.0337555)
Supplement: S1 Table — The table downward shows the descriptive statistics (Mean and N) of the variables used. (DOCX) [file pone.0337555.s001.docx]

**S1 Table. Descriptive Statistics.**

The table downward shows the descriptive statistics (Mean and N) of the variables used.

| Economy | AGE | gender(1=male,0=female) | education years | DEGREE | individual income in own currenty | family income in own currency | log of 5-year GDP per capita at age 15 |
| --- | --- | --- | --- | --- | --- | --- | --- |
| Austria | 45.1 | 0.5 | 11.3 | 2.4 | 1236.9 | 2184.2 | 10.2 |
| N | 1019 | 1019 | 1019 | 1019 | 829 | 736 | 973 |
| Australia | 52.5 | 0.4 | 13.5 | 3.5 | 39946.5 | 60502.0 | 10.1 |
| N | 1484 | 1494 | 1478 | 1471 | 1390 | 1337 | 1354 |
| Belgium | 48.9 | 0.5 | 12.8 | 2.9 | 1513.0 | 2785.0 | 10.0 |
| N | 1115 | 1115 | 1109 | 1115 | 1077 | 1009 | 1022 |
| Switzerland | 50.1 | 0.4 | 11.7 | 2.6 | 4241.0 | 6993.7 | 10.8 |
| N | 1229 | 1229 | 1225 | 1229 | 935 | 873 | 1088 |
| China Mainland | 43.0 | 0.5 | 8.2 | 2.2 | 13951.7 | 30708.0 | 7.7 |
| N | 3010 | 3010 | 3005 | 3005 | 2942 | 2850 | 2958 |
| Germany | 49.6 | 0.5 | 11.2 | 2.2 | 1328.5 | 2410.8 | 10.1 |
| N | 1388 | 1395 | 1393 | 1385 | 1244 | 1167 | 1257 |
| Denmark | 50.2 | 0.5 | 13.6 | 3.4 | 287702.1 | 509768.1 | 10.2 |
| N | 1518 | 1518 | 1429 | 1518 | 1460 | 1423 | 1403 |
| Spain | 47.1 | 0.5 | 12.2 | 2.6 | 987.4 | 1440.5 | 9.8 |
| N | 1215 | 1215 | 1152 | 1204 | 696 | 713 | 1120 |
| Finland | 47.6 | 0.5 | 14.5 | 3.0 | 2406.9 | 4878.1 | 9.8 |
| N | 879 | 880 | 813 | 856 | 799 | 768 | 874 |
| France | 55.1 | 0.5 | 13.7 | 2.8 | 1702.8 | 3015.9 | 9.9 |
| N | 2817 | 2817 | 2709 | 2792 | 2503 | 2605 | 2484 |
| U.K. | 50.4 | 0.4 | 12.5 | 2.5 | 13361.2 | 28313.5 | 10.0 |
| N | 852 | 853 | 852 | 795 | 814 | 756 | 773 |
| Italy | 48.3 | 0.5 | 14.6 | 3.0 | 1160.4 | 2288.9 | 10.0 |
| N | 1055 | 1070 | 1005 | 1074 | 950 | 960 | 992 |
| Japan | 49.2 | 0.5 | 12.8 | 3.2 | 2645069.0 | 5962821.0 | 9.6 |
| N | 1296 | 1296 | 1165 | 1284 | 1227 | 1170 | 1212 |
| South Korea | 43.5 | 0.5 | 12.9 | 3.4 | 2433099.0 | 3924449.0 | 8.8 |
| N | 1593 | 1599 | 1596 | 1596 | 952 | 1551 | 1506 |
| Norway | 48.0 | 0.5 | 14.6 | 3.6 | 625250.2 | 1190832.0 | 10.4 |
| N | 1246 | 1246 | 1158 | 1225 | 1207 | 1183 | 1193 |
| New Zealand | 50.4 | 0.4 | 13.9 | 3.3 | 43505.8 | 70968.8 | 10.0 |
| N | 890 | 896 | 893 | 888 | 860 | 849 | 816 |
| Portugal | 49.4 | 0.4 | 9.1 | 2.0 | 694.4 | 1140.7 | 9.5 |
| N | 1000 | 1000 | 996 | 1000 | 593 | 571 | 903 |
| Sweden | 48.5 | 0.5 | 12.6 | 3.0 | 23442.0 | 40879.1 | 10.1 |
| N | 1137 | 1137 | 1075 | 1118 | 1013 | 1019 | 1088 |
| Taiwan District | 44.8 | 0.5 | 11.7 | 2.9 | 28799.3 | 73971.1 | 9.0 |
| N | 2026 | 2026 | 2023 | 2026 | 2003 | 1905 | 1898 |
| U.S. | 49.5 | 0.5 | 13.6 | 3.7 | 26299.0 | 58076.7 | 10.3 |
| N | 1561 | 1581 | 1580 | 1580 | 1506 | 1444 | 1418 |
| Total | 48.5 | 0.5 | 12.3 | 2.9 | 280763.7 | 632520.0 | 9.6 |
| N | 28330 | 28396 | 27675 | 28180 | 25000 | 24889 | 26332 |
